# Illuminating Siderophore Transporter Functionality with Thiopeptide Antibiotics

**DOI:** 10.1128/mbio.03326-22

**Published:** 2023-03-22

**Authors:** Stephen K. Dolan

**Affiliations:** a School of Biological Sciences, Georgia Institute of Technology, Atlanta, Georgia, USA; b Emory Children’s Cystic Fibrosis Center, Atlanta, Georgia, USA; c Center for Microbial Dynamics and Infection, Georgia Institute of Technology, Atlanta, Georgia, USA

**Keywords:** *Pseudomonas aeruginosa*, fungal-bacterial interactions, iron acquisition, membrane transport, siderophores

## Abstract

The Gram-negative opportunistic pathogen Pseudomonas aeruginosa is a leading cause of infections and mortality in immunocompromised patients. This organism can overcome iron deprivation during infection via the synthesis of two iron-chelating siderophores, pyoverdine and pyochelin, which scavenge iron from host proteins. P. aeruginosa can also uptake xenosiderophores produced by other bacteria or fungi using dedicated transporter systems. The precise substrate specificity of these siderophore transporters remains to be determined. The thiopeptide antibiotic thiostrepton exploits the pyoverdine transporters FpvA and FpvB to cross the outer membrane and reach intracellular targets. Using a series of intricate biochemical experiments, a recent study by Chan and Burrows capitalized on the specificity of thiostrepton to uncover that FpvB transports the xenosiderophores ferrichrome and ferrioxamine B with higher affinity than pyoverdine. This surprising result highlights an alternative uptake pathway for these siderophores and has significant implications for our understanding of iron acquisition in this organism.

## COMMENTARY

Pseudomonas aeruginosa is a metabolically versatile bacterium that can cause devastating acute and chronic infections in immunocompromised individuals (1). Iron is essential for bacterial growth; however, mammals sequester iron using the iron-binding proteins transferrin and lactoferrin, which prevent infection through nutrient restriction, a concept known as nutritional immunity (2). When subjected to these host-mediated stresses, P. aeruginosa acquires iron using extracellular Fe^3+^ chelating small molecules termed siderophores (2). This bacterium produces two siderophores, pyoverdine and pyochelin, characterized by high and low affinities for iron, respectively (3). Iron-laden siderophores are then taken up by this bacterium via TonB-dependent transporters (TBDTs) (4). P. aeruginosa also encodes multiple TBDTs designed to “steal” structurally distinct iron-loaded siderophores produced by neighboring microorganisms (siderophore piracy) (5). TBDT proteins are thought to be quite specific to their respective substrates, and their expression is frequently orchestrated by a series of intricate feed-forward regulation loops (4, 6). Determining the ligand specificity of TBDTs is challenging. Although characterization has been aided by sequence alignments, proteomics, RT-PCR, and gene deletion studies, the expression of multiple, functionally redundant TBDTs can mask important phenotypes (7, 8).

TBDTs have 22-stranded β-barrels, with the lumen occluded by an N-terminal globular “plug” domain that contains a Ton box on the periplasmic side (4, 9). When the extracellular substrate binds, the plug domain becomes disordered, and this transition leads to a noncovalent complex between the plug domain and the C-terminal periplasmic domain of TonB. TonB forms a complex with the proteins ExbB and ExbD, and transport is mediated by utilizing energy derived from the proton motive force transmitted from the TonB−ExbB−ExbD complex located in the inner membrane. This TonB–Ton box interaction is finely tuned, facilitating the passage of a specific ligand by partially unfolding residues in the N-terminal plug subdomain to allow selective transport. This partial unfolding restricts the free diffusion of large antibiotics into the cell (4, 9).

The expression of TBDTs on the cell surface renders bacteria vulnerable to attack by antimicrobial compounds, bacteriophages, and bacteriocins, which exploit these transporters to bypass the outermost barrier of Gram-negative bacteria (10 to 13). Thiopeptides are a class of natural-product antibiotics that impact protein synthesis in target organisms. Over 100 thiopeptides with varied targets have been discovered to date (14). The majority of bacteria that encode thiopeptide biosynthetic gene clusters belong to either the actinobacteria or bacilli classes. Although the specific biological function of most thiopeptides has yet to be elucidated, under iron-limited conditions, the thiopeptide thiostrepton (TS) can cross the outer membranes of P. aeruginosa and Acinetobacter baumannii using pyoverdine-specific TBDTs (15). When starved of iron using iron chelators, P. aeruginosa increases pyoverdine receptor expression, allowing a greater influx of TS into the cell. In support of this hypothesis, pronounced synergy was observed between iron chelators and TS. Two additional thiopeptides, thiocillin and micrococcin, were shown to enter P. aeruginosa through a different TBDT siderophore transporter, specific to ferrioxamine (FiuA) (11). There also appear to be interspecies differences in thiopeptide susceptibility, suggesting that thiopeptide structures may have been shaped by evolution to target TBDTs of specific species (11, 14 to 16).

In a recent article in *mBio*, Chan and Burrows presented an important series of experiments using the thiopeptide TS to probe the specificity of siderophore transporters in P. aeruginosa, with unexpected results (17). TS crosses the outer membrane of P. aeruginosa through the primary pyoverdine transporter FpvA. TS can also use FpvB, which is thought to be a secondary transporter for pyoverdine. Accordingly, P. aeruginosa TS susceptibility required the presence of either FpvA or FpvB, and a Δ*fpvA*Δ*fpvB* double mutant proved to be TS resistant. A Δ*fpvA* mutant, expressing FpvB alone remained sensitive to TS (17).

The addition of pyoverdine-Fe^3+^ to a TS inhibition assay antagonizes TS uptake, rescuing P. aeruginosa growth in the presence of this antimicrobial (15). This is likely mediated by competition of the iron-loaded siderophore with TS for the same binding site on the FpvA and FpvB transporters. Other iron-loaded siderophores did not reduce TS susceptibility. Surprisingly, in a Δ*fpvA* mutant with FpvB alone expressed, pyoverdine does not antagonize TS uptake (17). This suggests that TS binds to FpvB with a higher affinity than pyoverdine. Could this mean that pyoverdine is not the preferred substrate of FpvB? Indeed, ferrichrome and, to a lesser extent, ferrioxamine B antagonized TS activity in a Δ*fpvA* mutant. With additional control and uptake experiments, the authors convincingly conclude that FpvB is a poor pyoverdine transporter that preferentially transports the xenosiderophores ferrichrome and ferrioxamine B. Interestingly, ferrichrome, produced by fungi, has a dedicated transporter in P. aeruginosa, FiuA, and ferrioxamine B, produced by *Streptomyces* spp., is transported by FoxA (18, 19).

Remarkably, FpvB variants (R191A and D219A) were generated that resulted in the inability of ferrichrome supplementation to antagonize TS activity. However, these FpvB variants still supported the growth of a P. aeruginosa siderophore-null mutant in the presence of ferrichrome-Fe^3+^ as an iron source. This suggests that these mutations specifically reduce the affinity of FpvB for ferrichrome, while still permitting TS uptake.

Finally, the authors use a combination of structural modeling, site-directed mutagenesis, and an intricate whole-cell sensor fluorescence-quenching assay to determine the affinity of siderophores for FpvA and FpvB, and their putative ligand binding sites (17). In this fluorescence assay, siderophore binding triggers conformational changes at the extracellular loops of the TBDT, leading to fluorophore quenching. Fluorescence recovery occurs once the siderophore is taken up and the loop returns to its original conformation. Unexpectedly, the quenching curve of ferrioxamine B-Fe^3+^ supported a two-site binding model. This result suggests that the binding mode of ferrioxamine B for FpvB is different than those of ferrichrome and pyoverdine; different ligands can interact in distinct ways with the same TBDT. Ferrioxamine B and E were reported to bind their primary transporter FoxA only at a single site (7, 20). Further experiments uncovered specific mutations (R191A and W347V) that abolished the initial quenching event observed for WT FpvB without impacting the second binding event. These data support that ferrioxamine B binds to FpvB at two distinct sites on this transporter, and that conformational changes from the first binding event appear to be independent of the second (17).

Thiopeptide-based screening assays may be an effective strategy to investigate the substrate specificity of uncharacterized TBDT systems in P. aeruginosa and other multidrug-resistant opportunistic pathogens. As well as those with defined roles in siderophore import, there are a total of ~35 predicted TBDTs encoded by P. aeruginosa (21). These are thought to transport crucial ligands, including cobalamin, zincophores, and other metal complexes (21, 22). Which of these other TBDTs are highly expressed by P. aeruginosa during growth in clinically relevant contexts? Are the sequences of these TBDTs conserved across P. aeruginosa clinical and environmental isolates?

A key hurdle will be to discover thiopeptides with novel TBDT specificity, although bioinformatic predictions suggest that a myriad of thiopeptides remain uncharacterized (23). Importantly, the screening for thiopeptide activity should be carried out when the target organism is cultured under clinically relevant conditions (such as micronutrient limitation) to maximize the translational potential of these findings. This work also provides a clear framework for the iterative synthesis and testing of novel thiopeptide-like or siderophore-antibiotic conjugates with targeted activity against P. aeruginosa or other organisms. Cocrystal structures of thiopeptides with their respective transporters would be an asset to advance this aim. The ability of numerous structurally diverse siderophores to interact at multiple, distinct sites within a single TBDT like FpvB is surprising, but this may turn out to be a widespread trend as additional siderophore TBDTs are examined in detail.

Another key finding of this work is that P. aeruginosa encodes two dedicated transport systems for the uptake of both ferrichrome and ferrioxamine B. This suggests that these siderophores are a valuable source of iron in the natural niche of this pathogen. The siderophore content in soil ranges between 2 and 279 nM, with ferrichrome and ferrioxamine-type siderophores being the most abundant (24). Therefore, the substrate specificity of FpvB most likely evolved to exploit this abundant source of free, siderophore-chelated iron. However, the importance of xenosiderophore utilization in P. aeruginosa has yet to be fully established. In a mouse model of infection, fungal gut microbiota and fungi present in the diet have recently been shown to produce the siderophores ferrichrome and coprogen (25). Ferrichrome cannot be bound by the siderophore-binding host protein lipocalin-2, so it may be a valuable source of iron during infection. These siderophores conferred a competitive growth advantage to the intestinal pathogen Salmonella enterica serovar Typhimurium expressing the TDBTs FhuA and FhuE. The competitive advantage of strains expressing FhuA and FhuE was greater when inflammation levels in mice increased (25).

Considering the high incidence of both ferrichrome and ferrioxamine-type siderophores in polymicrobial environments, and the widespread, cross-kingdom pirating of these molecules, it is tempting to speculate that the feed-forward regulation of xenosiderophore TBDT expression may have been shaped by antimicrobial compounds like thiopeptides. It may be risky for P. aeruginosa to depend entirely on crude environmental cues like low iron to control the expression of xenosiderophore uptake systems, as this would provide TBDT-dependent antimicrobials with an unrestricted pathway into the cell. This presents a remarkable contrast, as we utilize thiopeptides to illuminate the functionality of transporters, which were presumably shaped by evolution to hide from these potent antimicrobials.
